# Effect of Cu^2+^ on Corrosion Behavior of A106B Carbon Steel and 304L Stainless Steels in Seawater

**DOI:** 10.1155/2021/6661872

**Published:** 2021-10-06

**Authors:** Kewei Fang, Chengtao Li, Shuai Dong, Dubao Zhang, Xiangfeng Wu, Hongxiang Hu

**Affiliations:** ^1^Suzhou Nuclear Power Research Institute, Suzhou 215004, China; ^2^CAS Key Laboratory of Nuclear Materials and Safety Assessment, Institute of Metal Research, Chinese Academy of Sciences, Shenyang 110016, China

## Abstract

The corrosion behaviors of A106B carbon steel and 304L stainless steel (SS) in seawater with different Cu^2+^ concentrations were studied by the immersion test and the potentiodynamic polarization test. The results showed that with the increasing Cu^2+^ concentration, the mass lot rates of A106B and 304L SS all increased in the immersion test, and compared with A106B, the mass lot rates of 304L SS were all smaller. In the potentiodynamic polarization test, following the concentration of Cu^2+^ increased, the corrosion potential of A106B firstly shifted negatively; then, when Cu^2+^ increased to 100 ppm, the polarization curve moved to the upper right direction; namely, both the corrosion potential and corrosion electrical density increased. The corrosion potential of 304L SS increased with the increasing Cu^2+^, and the passive region was reduced; the pitting sensitivity improved.

## 1. Introduction

The important plant water system (SEC system), i.e., the safety plant water system, function was to transfer the heat from the structures, systems, and components related to safety to the final sink-seawater under normal operation and accident conditions. The system consisted of SEC pumps, shellfish traps, various values and pipes, and RRI/SEC plate heat exchangers [1].

In the existing nuclear power plants, the SEC pipes were made of stainless steel [2, 3], carbon steel lined with coating, rubber, plastic or cement mortar, and resin pipes. During the service of the SEC pipes, the addition of the cupric fungicide to prevent microbial corrosion, the corrosion of copper components, or other reasons could lead to the local enrichment of Cu^2+^ in the SEC system and the precipitation of Cu on the surface of components; the interaction between Cu^2+^ and seawater may accelerate the corrosion of SEC system material, even leading to the breaking of the components and inducing the addition of Cu^2+^ into the secondary circuit system.

Presently, the corrosion of steels in seawater had been widely investigated all over the world [4–20], and the influence of Cu^2+^ on material corrosion is mainly focused on different materials in nonseawater environment, such as steel [21–30], aluminum alloy [31, 32], 690 alloy [33], and copper alloy [34]. However, the works focused on the corrosion behavior of steels in seawater containing Cu^2+^ and the influence of the Cu^2+^ and copper oxides on the corrosion of the equipment in the secondary circuit system were little reported [35]. Therefore, the corrosion behaviors of steels in seawater containing Cu^2+^ were studied by the immersion test, potentiodynamic polarization, and SEM observation, which could provide a certain basis and guidance for the operation of nuclear power stations.

## 2. Experimental Detail

**Figure 1:**
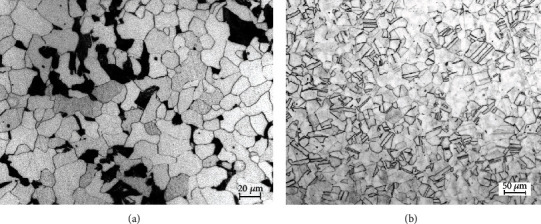
OM microstructure of (a) A106B and (b) 304L SS.

The materials used in the present work were A106B carbon steel and 304L stainless steel (SS). The chemical compositions (wt.%) of A106B and 304L SS are listed in Table 1. As shown in Figure 1(a), the optical microstructure (OM) of A106B was ferrite and pearlite biphasic structure, among which the black lamellar structure was pearlite phase. The microstructure of 304L SS is listed in Figure 1(b), which was a typical austenitic structure with twins. A106B was eroded by 4% nitrate alcohol and ethanol, respectively; then, they were analyzed by an optical microscope. 304L SS was electrolytically etched in 10% oxalic acid reagent at 10 V for 60 s for the microstructural observations.

The materials were cut into sheets with a dimension of 20 mm × 20 mm × 3 mm. Prior to the experiment, the surface of the samples was polished to 800# with sandpaper, then cleaned with deionized water, acetone, and anhydrous ethanol, then air-dried. The quality was weighted and size measured after the samples. The test solutions were seawater solutions with different concentrations of Cu^2+^; the concentrations of Cu^2+^ were 10, 50, 100, 500, and 1000 ppm, respectively; the test period was 168 h, and the test temperature was 50°C. Before and after the immersion tests, the specimen was weighed using an electronic balance with an accuracy of 0.01 mg. The corrosion rate (*C*_*R*_) was calculated based on the mass loss, Δ*m*, using Equation (1) after a given period [1]:

(1) $$\begin{array}{c} C_{R}=\frac{\Delta m_{1}+\Delta m_{2}+\Delta m_{3}+\Delta m_{4}+\Delta m_{5}}{5\cdot S\cdot t}, \end{array}$$

where Δ*m*_1_, Δ*m*_2_, Δ*m*_3_, Δ*m*_4_, and Δ*m*_5_ represent the mass loss of the five samples used (mg), *S* is the surface area of the sample (10.4 cm^2^), and *t* is the total test duration (168 h).

The exposed area of the sample in the test of the potentiodynamic polarization was 1 cm^2^. Before the experiment, the surface of the samples was polished down to 800# with sandpaper and then cleaned using deionized water, acetone, and anhydrous ethanol. The potentiodynamic polarization test was carried out using the CS310 electrochemical workstation; the detailed introduction of the electrochemical experimental design methods was presented in the literature [1, 2]. The test solution was consistent with the immersion test, and the test temperature was 25°C. Before the test, the working electrode was immersed in the solution for 30 min and then −400 mV below the corrosion potential and terminated when the current density of 10 mA/cm^2^ was reached with a scanning rate of 20 mV/min.

## 3. Results and Discussion

### 3.1. Corrosion Morphologies of A106B

**Figure 2:**
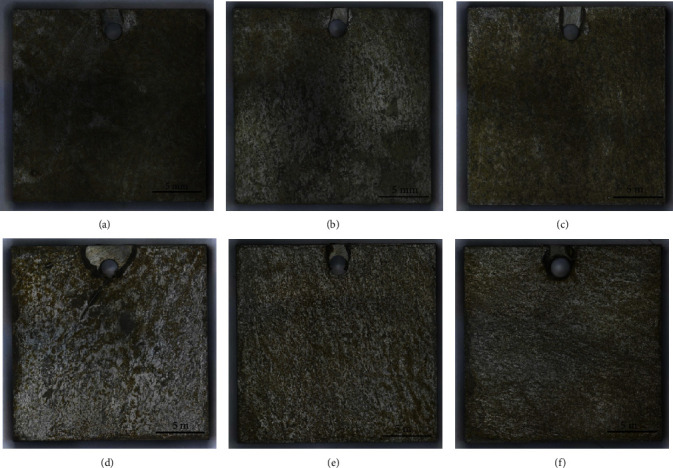
Corrosion morphologies of A106B after the immersion test: (a) 0 ppm, (b) 10 ppm, (c) 50 ppm, (d) 100 ppm, (e) 500 ppm, and (f) 1000 ppm.

Figure 2 presents the corrosion morphologies of A106B after the immersion test in tested solutions. A106B presented uniform corrosion in seawater of different concentrations of Cu^2+^. After the test in solution without Cu^2+^, the matrix surface of the sample was relatively flat; with the addition of Cu^2+^, small and shallow pits appeared, and with the increase of the concentration of Cu^2+^, the amount of the pits increased. After the test, a small number of tan corrosion products adhered to the matrix surface.

**Figure 3:**
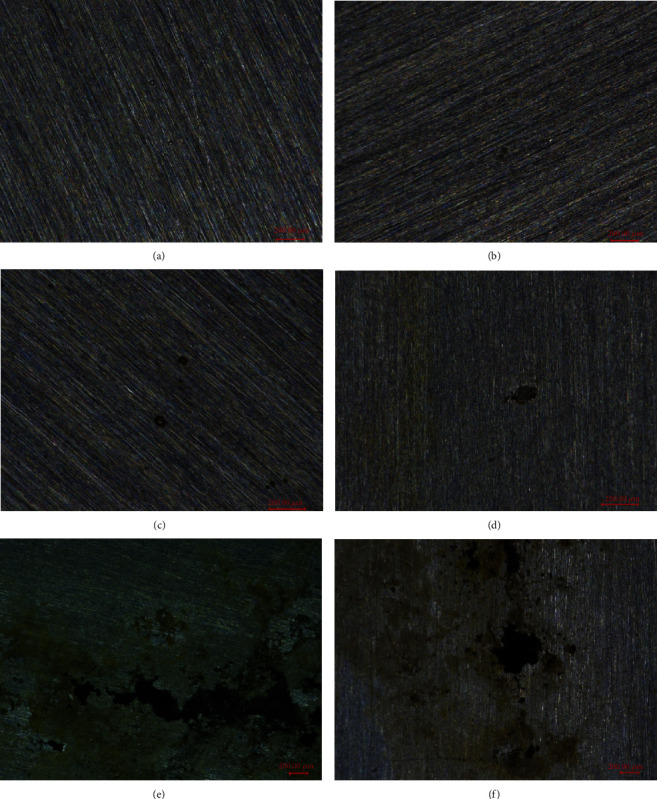
Morphologies of 304L SS after the immersion test: (a) 0 ppm, (b) 10 ppm, (c) 50 ppm, (d) 100 ppm, (e) 500 ppm, and (f) 1000 ppm.

**Figure 4:**
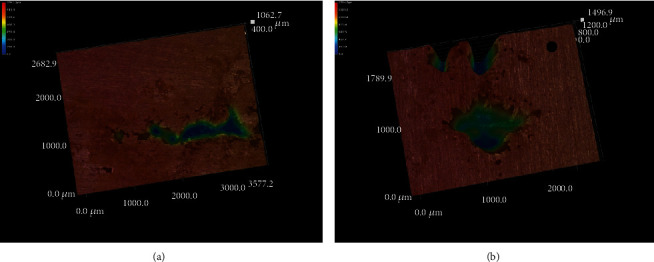
3D corrosion pit morphologies of 304L SS: (a) 500 ppm and (b) 1000 ppm.

Figures 3 and 4 present the corrosion morphologies of 304L SS after the immersion test. The corrosion pits all appeared on the matrix surface of the samples; without the Cu^2+^, there were only few shallow pits, and the size of the pits were small, with a few microns; with the increase of the concentration of Cu^2+^, the amount and size of the pits both increased; the maximum depth of the pits in seawater of 500 ppm and 1000 ppm Cu^2+^ could be as deep as 1000 and 1500 microns, respectively.

### 3.2. Corrosion Rate of A106B and 304L SS

**Figure 5:**
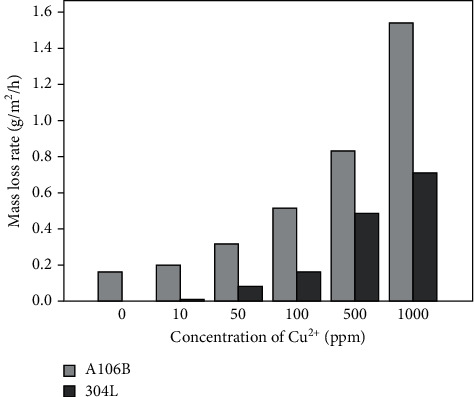
The weight loss rate of A106B and 304L SS after the immersion test.

Figure 5 presents the mass loss rate of A106B and 304L SS after the immersion test. As is seen in Figure 5, the mass loss rate of A106B largely increased with the increase of the concentration of Cu^2+^. The mass loss rate in seawater without the Cu^2+^ was 0.163 g·m^–2^·h^–1^; the mass loss rate in seawater of 10, 50, 100, 500, and 1000 ppm Cu^2+^ increased by 0.23, 0.97, 2.14, 4.12, and 8.47 times, respectively. The mass loss rate of 304L SS in seawater without the Cu^2+^ is about 0.0012 g·m^–2^·h^–1^. However, the mass loss rate in seawater of 10 ppm, 50 ppm, 100 ppm, 500 ppm, and 1000 ppm Cu^2+^ increased by 6, 67, 139, 404, and 591 times, respectively. The presence of Cu^2+^ significantly increased the corrosion rate of 304L SS in seawater.

### 3.3. Corrosion Morphologies of A106B and 304L SS

**Figure 6:**
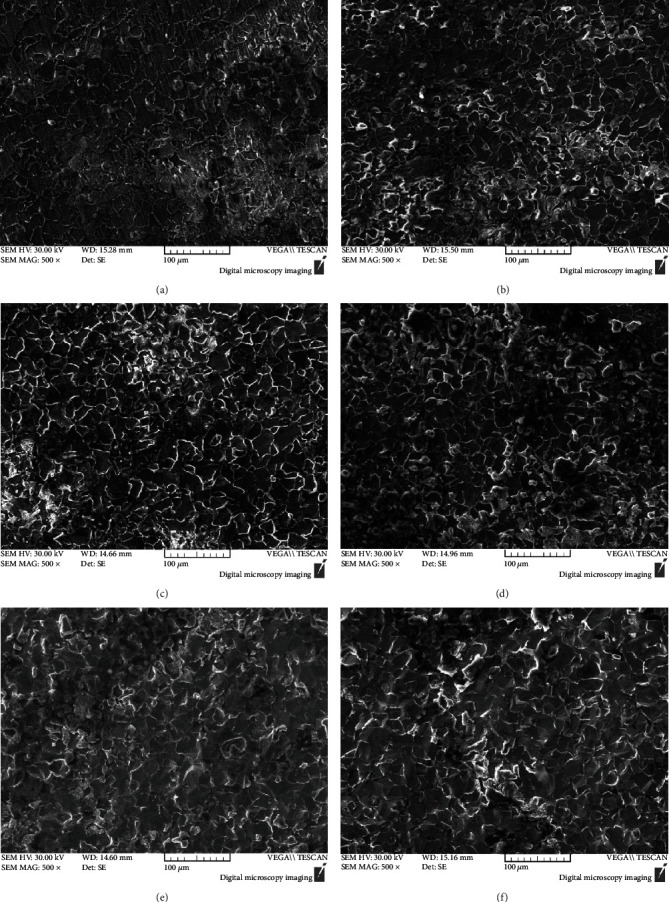
Morphologies of A106B after the immersion test: (a) 0 ppm, (b) 10 ppm, (c) 50 ppm, (d) 100 ppm, (e) 500 ppm, and (f) 1000 ppm.

**Figure 7:**
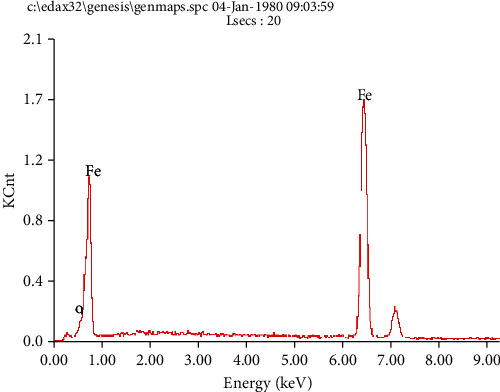
EDS on the surface of A106B after the immersion test at 100 ppm Cu solution.

Figure 6 presents the SEM surface morphologies of A106B after the immersion test in seawater solution of different concentrations of Cu^2+^. The morphologies of different concentrations of Cu^2+^ had no significant difference; the matrix was relatively flat with some small particles. The EDS analysis showed that the matrix surface mainly consisted of Fe and a small amount of O, the content of Cu was little, which indicated that the precipitated Cu and other corrosion products were very loose, and there were basically no residual corrosion products attached on the matrix.  Figure 7 presents the EDS result on the surface of A106B, which indicated that the surface of A106B was rich in Fe and O elements.

**Figure 8:**
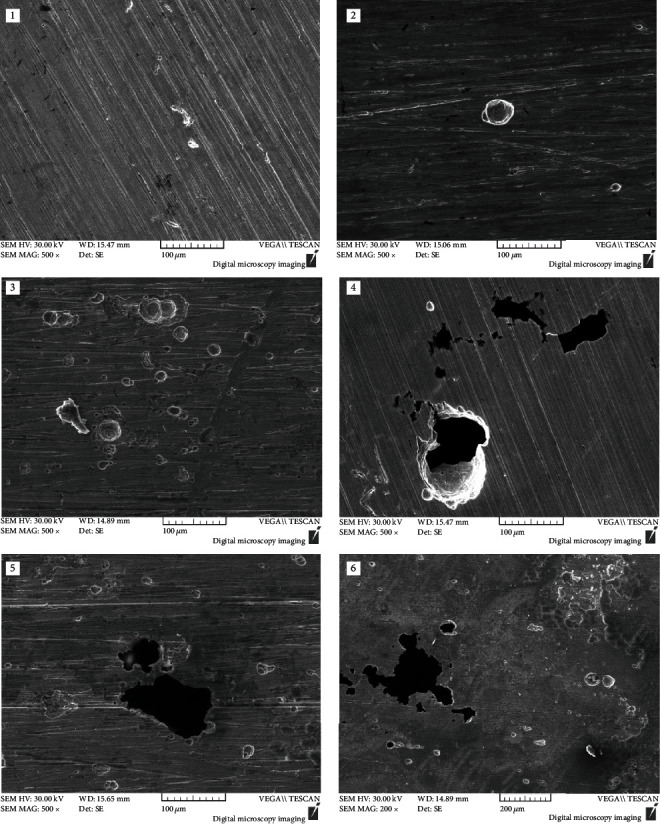
Morphologies of 304L SS after the immersion test: (1) 0 ppm; (2) 10 ppm; (3) 50 ppm; (4) 100 ppm; (5) 500 ppm; (6) 1000 ppm.

Figure 8 shows the SEM microscopic morphology of 304L stainless steel after the corrosion test in seawater of different concentrations of Cu^2+^. Without the Cu^2+^, the pitting corrosion initiation formed, the concentration of Cu^2+^ increased to 10 ppm, and a small number of corrosion pits with a few microns to tens of micron in size appeared; with the increase of the concentration of Cu^2+^, the size of the corrosion pits increased; when the concentration of Cu^2+^ increased to 100 ppm, the size of the corrosion pits had been more than 100 microns, which indicated the sample suffered from heavy pitting corrosion.

### 3.4. Potentiodynamic Polarization

**Figure 9:**
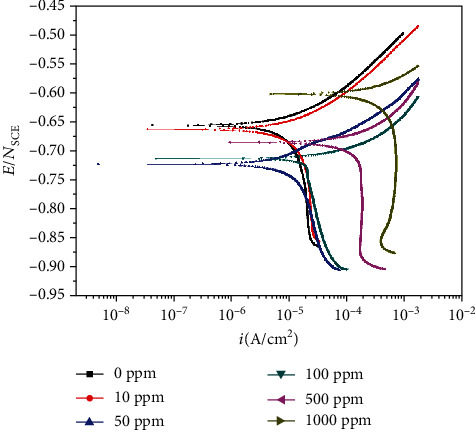
Potentiodynamic polarization curves of A106B in tested solutions.

**Figure 10:**
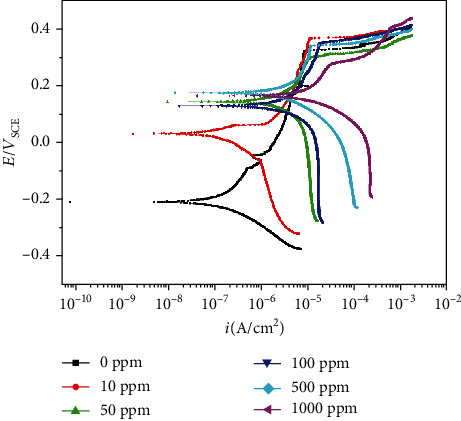
Potentiodynamic polarization curves of 304L SS in tested solutions.

Figure 9 shows the potentiodynamic polarization curves of A106B in tested solutions. As is seen in Figure 10, the concentration of Cu^2+^ had a significant influence on the corrosion behavior of A106B. With the increase of the concentration of Cu^2+^, the corrosion potential decreased and the corrosion current density increased firstly; compared with that without Cu^2+^, *E*_corr_ of A106B in tested solutions initially shifted to a more negative potential and then subsequently increased to negative potential when the concentration of Cu^2+^ is higher than 100 ppm. The corrosion potential (*E*_corr_) for A106B in seawater without Cu^2+^ is -648 mV_SCE_; when the concentration of Cu^2+^ increased to 10 ppm, *E*_corr_ decreased by about 10 mV; when increased to 50 ppm, it decreased by 65 mV_SCE_; when increased more than 100 ppm, the potentiodynamic polarization curves moved significantly up to the right; namely, both the corrosion potential and the corrosion current density largely increased. Meanwhile, with the increase of the concentration of Cu^2+^, the cathode control became the diffusion control of Cu^2+^.

The corrosion potential was a mixed potential formed by the coupling of anodic dissolution reaction and cathode depolarizer reduction reaction, which was between the anodic reaction equilibrium potential and cathode reduction reaction equilibrium potential. In seawater solution without Cu^2+^, the cathode reaction was the reduction of O_2_, as the equilibrium potential of electrode reaction Cu⇌Cu^2+^ + 2e^−^ was higher than that of Fe⇌Fe^2+^ + 2e^−^; when the Cu^2+^ was added, the cathode reactions on the surface of A106B included both the reduction reactions of O and Cu^2+^; namely, in the seawater containing Cu^2+^, there were two depolarizing agents, O_2_ and Cu^2+^, which made the A106B suffer from corrosion.

The equilibrium potential (for standard hydrogen potential) of electrode reaction Cu⇌Cu^2+^ + 2e^−^ could be calculated through the Nernst equation:

(2) $$\begin{array}{c} E_{\text{e}\left(\text{Cu}/{\text{Cu}}^{2+}\right)}={E^{\theta }}_{\left(\text{Cu}/{\text{Cu}}^{2+}\right)}+\frac{RT}{2F}\ln c_{{\text{Cu}}^{2+}}, \end{array}$$

where *E*_e(Cu/Cu^2+^)_ is the equilibrium potential of electrode reaction Cu⇄Cu^2+^ + 2e and *E*^*θ*^_Cu/Cu^2+^_ is the standard potential of electrode reaction Cu⇄Cu^2+^ + 2e, where *R* is ideal gas constant, 8.314 J/(K·mol), *T* is thermodynamic temperature (K), *F* is Faraday constant, 96500 C, and *c*_*Cu*^2+^_ is the concentration of Cu^2+^ (mol/cm^3^).

As known, *E*^*θ*^_Cu/Cu^2+^_ was known as 0.345 V (SHE), *E*^*θ*^_OH^−^/O_2__ was 0.401 V (SHE), so when the concentration of Cu^2+^ was low, the cathode reaction was mainly the reduction of O_2_; the two intercoupling cathode reactions accelerated the anodic dissolution reaction rate; meanwhile, a small amount of Cu was precipitated on the surface of the sample, which formed the Fe-Cu corrosion galvanic cells and also accelerated the anodic dissolution reaction. With the increase of the concentration of Cu^2+^, *E*_e(Cu/Cu^2+^)_ increased constantly and finally increased higher than *E*^*θ*^_OH^−^/O_2__, the cathode reaction was changed from mainly the reduction of O_2_ to mainly the reduction of Cu^2+^, a large amount of Cu was precipitated on the surface, and a large number of Fe-Cu corrosion galvanic cells were formed, which largely increased the anodic dissolution rate. This was also the reason why there were many small corrosion pits on the surface, but the macroscopic corrosion morphology was characterized by uniform corrosion.

Figure 10 shows the potentiodynamic polarization curves of 304L SS in tested solutions. As can be seen, with the increase of the concentration of Cu^2+^, the potentiodynamic polarization curves moved up to the right; the corrosion potential and corrosion current density increased. Due to the existence of the pass passivation film, the anodic dissolution rate was quite low and the anode reaction equilibrium potential had no significant change; with the increase of the concentration of Cu^2+^, the cathode reaction rate was accelerated and the cathode reaction equilibrium potential increased, and an anode reaction and two cathodic reactions coupled and polarized each other, finally leading to the increase of the corrosion potential and corrosion current density. Meanwhile, a small amount of Cu was precipitated on the surface of the sample, which also formed the Fe-Cu galvanic cells, making the stability of the passivation film decreased; the more the number of Fe-Cu galvanic cells increased, the more obvious the stability of the passivation film decreased; thus, the passivation zone significantly narrowed, and the pitting corrosion sensitivity *e* significantly increased; once the passivation film was broken locally, the fresh metal substrate was exposed; the corrosion pits formed and developed under the combined action of Cl^−^ and Cu^2+^.

## 4. Conclusion

1. The results of the immersion test showed that A106B presented uniform corrosion in seawater of different concentrations of Cu^2+^; with the increase of the concentration of Cu^2+^ to 10 ppm, 50 ppm, 10 0 ppm, 500 ppm, and 1000 ppm, the corrosion weight loss rate increased by 0.23, 0.97, 2.14, 4.12, and 8.47 times, respectively
2. 304L SS presented pitting corrosion in seawater of different concentrations of Cu^2+^; with the increase of the concentration of Cu^2+^ to 10 ppm, 50 ppm, 100 ppm, 500 ppm, and 1000 ppm, the corrosion weight loss rate increased by 6, 67, 139, 404, and 591 times, respectively; the amount and size of the corrosion pits both increased. Compared with the behaviors of A106B in the immersion test, the mass lot rates of 304L SS were all lower
3. The results of potentiodynamic polarization curves of A106B showed that with the increase of the concentration of Cu^2+^, the corrosion potential decreased firstly; when increased to more than 100 ppm, both the corrosion potential and the corrosion current density largely increased. Meanwhile, the cathode reaction was altered from mainly the reduction of O_2_ to mainly the reduction of Cu^2+^
4. The results of potentiodynamic polarization curves of 304L showed that with the increase of the concentration of Cu^2+^, the corrosion potential and corrosion current density increased, the passivation zone significantly narrowed, and the pitting corrosion sensitivity significantly increased

## Figures and Tables

**Table 1:** The chemical compositions of A106B and 304L SS (wt.%).

	C	Si	Mn	P	S	Cr	Ni	Mo	Cu	Fe
A106B	0.23	0.35	0.62	0.023	0.012	0.21	0.14	0.07	0.18	Bal.
304L SS	0.025	0.27	1.38	0.017	0.002	18.04	8.08	0.05	0.07	Bal.

## Data Availability

The data used to support the findings of this study are available from the corresponding authors upon request.

## References

[1] Li L. L., Wang Z. B., He S. Y., Zheng Y. G. (2021). Correlation between depassivation and repassivation processes determined by single particle impingement: its crucial role in the phenomenon of critical flow velocity for erosion-corrosion. *Journal of Materials Science & Technology*.

[2] Zhang L. M., Li Z. X., Hu J. X. (2021). Understanding the roles of deformation-induced martensite of 304 stainless steel in different stages of cavitation erosion. *Tribology International*.

[3] Ju H., Xu W. H., Chen J. J., Zhang D. L., Liu G. M., Duan J. Z. (2020). Electrochemical investigation of under-deposit corrosion behavior for aluminum brass in artificial seawater. *Corrosion*.

[4] Tian H. Y., Chen M. D., Ge F., Song K., Wang X., Cui Z. Y. (2021). Hydrogen permeation and stress corrosion cracking of heat-affected zone of E690 steel under the combined effect of sulfur species and cathodic protection in artificial seawater. *Construction and Building Materials*.

[5] Qiao Y. X., Sheng S. L., Zhang L. M. (2021). Friction and wear behaviors of a high nitrogen austenitic stainless steel Fe-19Cr-15Mn-0.66N. *Journal of Mining and Metallurgy, Section B: Metallurgy*.

[6] Qiao Y. X., Chen Y. P., Li L. L. (2021). Corrosion behavior of a nickel-free high-nitrogen stainless steel with hydrogen charging. *JOM*.

[7] Shen Y. Y., Dong Y. H., Dong L. H., Yin Y. S. (2020). Corrosion inhibition effect of microorganism on 5754 Al alloy in seawater. *Aata Metallurgica Sinica*.

[8] Xu P. F., Feng X. G., Lu X. Y., Chen C. (2020). Effect of calcium phytate on the corrosion behavior of 304 stainless steel as coral concrete reinforcement in a 3.5% sodium chloride solution. *International Journal of Electrochemical Science*.

[9] Chen Z. W., Xia W. T., Yao C. Q., Lin Z. F., Zhang W., Li W. H. (2020). Research on the metal corrosion process in the sea mud/seawater/atmosphere interface zone. *Coatings*.

[10] Ogawa Y., Suzuki S., Taniguchi N., Kawasaki M., Suzuki H., Takahashi R. (2021). Corrosion resistance of a cast steel overpack for high-level radioactive waste disposal in Japan. *Materials and Corrosion*.

[11] Elshami A., Bonnet S., Khelidj A., Sail L. (2020). Effectiveness of corrosion inhibitors in simulated concrete pore solution. *European Journal of Environmental and Civil Engineering*.

[12] Chen Z. X., Hu H. X., Zheng Y. G., Guo X. M. (2021). Effect of groove microstructure on slurry erosion in the liquid-solid two- phase flow. *Wear*.

[13] Benea L., Simionescu N., Mardare L. (2020). The effect of polymeric protective layers and the immersion time on the corrosion behavior of naval steel in natural seawater. *Journal of Materials Research and Technology*.

[14] Wang D. P., Zhang H. T., Guo P. Y., Sun B. A., Wang Y. X. (2021). Nanoscale periodic distribution of energy dissipation at the shear band plane in a Zr-based metallic glass. *Scripta Materialia*.

[15] Kim Y. B., Kim S. J. (2019). Erosion corrosion characteristics of Al5052-O and Al6061-T6 aluminum alloys with flow rate of seawater. *Corrosion Science and Technology*.

[16] Gao L. F., Du M. (2017). Pitting corrosion behavior of 304 stainless steel in desalination seawater. *Corrosion Science and Protection Technology*.

[17] Chernov B. B., Chaves I. A., Nugmanov A. M., Melchers R. E. (2018). Corrosion performance of low alloy steels in sub-arctic natural seawater. *Corrosion*.

[18] Mao Y. Z., Wei Y. H., Zhao H. T., Lv C. X., Cao H. J., Li J. (2018). Corrosion behavior of epoxy-coated rebar with pinhole defect in seawater concrete. *Acta Metallurgica Sinica-Einglish Letters*.

[19] Zhang B. S., Dong Q., Ba Z., Zhu S., Han Y., Wang Z. (2018). Electrochemical corrosion behavior of plasma-sprayed FeCrNiMoCBSi amorphous/nanocrystalline coatings in simulated seawater medium. *Journal of Materials Engineering and Performance*.

[20] Ma F. L., Li J., Zeng Z., Gao Y. (2018). Tribocorrosion behaviour of F690 and 316L steel in artificial seawater. *Lubrication Science*.

[21] Aguirre J., Walczak M. (2017). Effect of dissolved copper ions on erosion-corrosion synergy of X65 steel in simulated copper tailing slurry. *Tribology International*.

[22] Qu Q., Gao G., Guo H., Li L., Ding Z. (2011). Co-effect of bis (cyclo-hexanone) oxalyldihydrazone and copper (II) ion on the corrosion of cold rolled steel in 0.5 M hydrochloric acid solution. *Materials and Corrosion*.

[23] Migahed M. A., Hegazy M. A., al-Sabagh A. M. (2012). Synergistic inhibition effect between Cu^2+^ and cationic gemini surfactant on the corrosion of downhole tubing steel during secondary oil recovery of old wells. *Corrosion Science*.

[24] Tanaka H., Miyafuji A., Ishikawa T., Nakayama T. (2018). Influence of Ni(II), Cu(II) and Cr(III) on the formation, morphology and molecular adsorption properties of *α*-FeOOH rust particles prepared by aerial oxidation of neutral Fe(II) solutions. *Advanced Powder Technology*.

[25] Li P. P., Zhang H. L., Xia M. Z., Wang F. Y., Zhu S. D., Lei W. (2019). The synergistic effect and microscopic mechanism of co-adsorption of three emerging contaminants and copper ion on gemini surfactant modified montmorillonite. *Ecotoxicology and Environmental Safety*.

[26] Li Q., Zhang D. P., Hu S., Chen Z. Y., Guo X. P. (2020). Effect of the ion selectivity of a precipitate membrane on the corrosion of carbon steel in Cu^2+^-containing solution. *Materials Chemistry and Physics*.

[27] Mu G. N., Zhao T. P., Liu M., Gu T. (1996). Effect of metallic cations on corrosion inhibition of an anionic surfactant for mild steel. *Corrosion*.

[28] Liu J., Alfantazi A., Asselin E. (2014). Influence of cupric, ferric, and chloride on the corrosion of titanium in sulfuric acid solutions up to 85°C. *Corrosion*.

[29] Fan L., Zhang J. T., Wang H. (2021). Effects of trace Cl^−^, Cu^2+^ and Fe^3+^ ions on the corrosion behaviour of AA6063 in ethylene glycol and water solutions. *Acta Metallurgica Sinica-English Letters*.

[30] Kameli D., Aliouane N., Hammache-Makhloufi H., Makhloufi L. (2020). Anti corrosion activity of ethylene tetra phosphonic acid-Cu^2+^ system on carbon steel in H2SO4SOLUTION. *Surface Review and Letters*.

[31] El Meleigy A. E. (2006). Influence of copper ions on the corrosion behaviour of aluminium. *Egyptian Journal of Chemistry*.

[32] Ahmed M., Qi Y. M., Zhang L. L. (2020). Influence of Cu^2+^ ions on the corrosion resistance of AZ31 magnesium alloy with microarc oxidation. *Materials*.

[33] Lin Z., Qiu S., Xiao J., Fu Z., Chen Y. (2015). Effects of Cl^−^ and Cu^2+^ on stress corrosion cracking of alloy 690. *Nuclear Power Engineering*.

[34] Giordano C. M., Duffó G. S., Galvele J. R. (1997). The effect of Cu^2+^ concentration on the stress corrosion cracking susceptibility of *α*-brass in cupric nitrate solutions. *Corrosion Science*.

[35] Aoyama T., Ogawa H., Kato C., Ueno F. (2021). Decrease in pitting corrosion resistance of extra-high-purity type 316 stainless-steel by Cu^2+^ in NaCl. *Metals*.

